# 2D electrophoresis image brightness correction based on gradient interval histogram

**DOI:** 10.1186/s12859-020-3432-y

**Published:** 2020-03-19

**Authors:** Qiaofeng Ou, Jiabing Xiao, Lei Yu, Kaizhi Wu, Bangshu Xiong

**Affiliations:** Key Laboratory of Image Processing and Pattern Recognition of Jiangxi Province, Nanchang Hangkong University, Nanchang, 330063 China

**Keywords:** Two-dimensional electrophoresis image, Protein spot, Gradient interval histogram, Spot quantification

## Abstract

**Background:**

Two-dimensional electrophoresis (2DE) is one of the most widely applied techniques in comparative proteomics. The basic task of 2DE is to identify differential protein expression by quantitative analysis of 2DE images. To reduce the errors of spot quantification in 2DE images, a novel brightness correction method based on gradient interval histogram (GIH) is proposed in this paper.

**Results:**

First, GIH equalization is proposed to enhance the protein spot edges, especially the weak protein spots in the 2DE image. Second, to eliminate the overall brightness shift, GIH matching is applied to the 2DE images that need to be compared. Finally, the proposed method is verified by subjective quality evaluation and quantitative analysis of protein spots in real 2DE images.

**Conclusions:**

The experimental results show that the average error of the quantification of corresponding protein spots in the resulting image pairs is less than 3%, which is significantly superior to that of the existing methods.

## Background

Two-dimensional electrophoresis (2DE) is one of the commonly used techniques for proteomics research [1–7]. The aim of 2DE image analysis is to identify differential protein expression, which is the basis for disease diagnosis and drug development. The identification accuracy relies on the spot quantification [8]. Due to the influence of complex electrophoresis experiments and image acquisition, a large number of low-abundance proteins are presented as weak spots that are difficult to detect. Moreover, among different 2DE images, there are usually overall shifts in brightness that add error of spot quantification. Therefore, brightness correction is a necessary preprocessing step in 2DE image analysis [9].

Researchers have proposed a variety of methods for 2DE image brightness correction [9–15]. In 2006, Kazhiyur-Mannar et al. [10] used contour wavelet filtering to alleviate the influence of inconsistent background brightness. For the time-frequency domain filtering method, adjusting parameters for different 2DE images is a complex task, and the computation is intensive. Hence, this method is still in the theoretical research stage. In 2007, Seller et al. [11] analyzed the effects of light and cameras on 2DE images. In 2008, Rye et al. [12] proposed to reduce noise and background inconsistency with image morphology, and the key aspect of the method was to choose an appropriate structural element. In 2016, Wu et al. [13] proposed a method of modeling brightness inhomogeneity by using polynomial fitting of slowly varying gradients and succeeded in removing some background intensities and weak protein spots simultaneously. Specific correction methods in other domains, such as those proposed in the references [14–16], are applicable to images with certain characteristics but are not ideal for 2DE images. In addition, as conventional enhancement methods, histogram equalization (HE) and contrast limited adaptive histogram equalization (CLAHE) involve over-enhancement [17].

The two goals of 2DE image brightness correction are as follows. The first is to reduce the miss rate of detection by enhancing the weak protein spots. The second is to reduce the error of spot quantification by adjusting the 2DE images to a similar average brightness.

The basic principle of HE is to generate a gray-level mapping curve according to the probability distribution of pixel brightness. The contrast gain is proportional to the height of the gray-level histogram. In 2DE images, the bright pixels in the background constitute the majority, and the dark pixels of distinct protein spots also account for a large proportion. Hence, large peaks in the histogram of a 2DE image can be caused by uninteresting areas (especially background and over-saturated protein spots). In this case, the enhancement gain of the background and over-saturated protein spots is excessive large, which is called as over-enhancement. In addition, the histogram reaches a minimum at the gray level of weak spots. Hence, the contrast gain of weak spots is the least. Therefore, the conventional HE method cannot satisfy the above two goals of brightness correction.

To avoid over-enhancement, CLAHE limits the local height of the histogram by cutting and redistributing the peaks of the gray histogram that exceed the given threshold. This method has achieved good application results in some fields [10, 11]. However, the clipped histogram peaks are redistributed by average to the whole grayscale, the histogram height of the weak protein spots increases very little, and the contrast gain of the weak protein spots remains small. Hence, CLAHE is also incapable of achieving the two goals of 2DE image brightness correction.

Therefore, a 2DE image brightness correction method based on the gradient interval histogram (GIH) is proposed in this paper. First, the GIH equalization (GIHE) algorithm is proposed to enhance the brightness of weak protein spots in reference 2DE images, and the overall brightness of the test images is adjusted by the GIH match (GIHM) method to reduce error of spot quantification, thus improving the identification accuracy of differential protein expression.

## Results

To verify the effectiveness and superiority of the proposed algorithm, we implemented the algorithm with C++ programming and tested real 2DE images. The tested 2DE images consist of real scanned 2DE images from our laboratory and the PDQuest test 2DE image database. Furthermore, the proposed method is compared with the morphological top-hat transformation method [8], 2D polynomial fit method [13], multi-scale retinex method [16] and CLAHE method [17].

### Experiments on the enhancement of weak protein spots based on GIHE

**Fig. 1:**
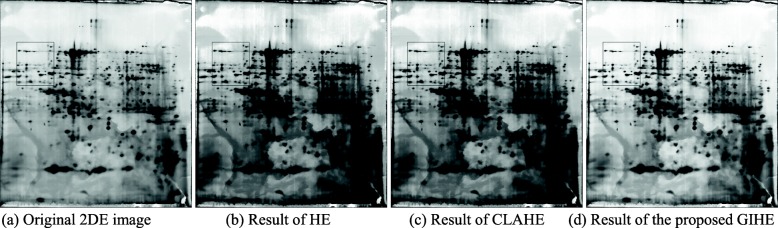
Brightness correction results of a real 2DE image

**Fig. 2:**
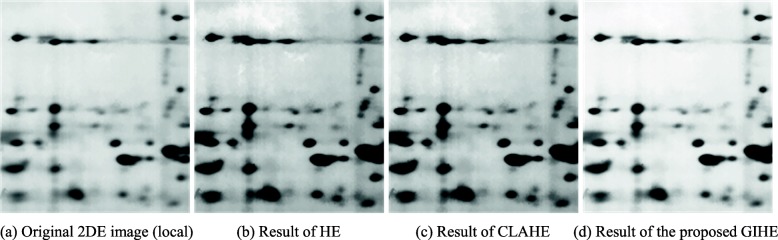
Enlarged image blocks in Fig. 1

Figure 1 shows the enhancement results of weak protein spots in a real 2DE image collected by our research group. Figure 1(a) shows the original real 2DE image from our laboratory. The background of the reference image is dark due to the residual gel stain, and there are a large number of weak protein spots in the upper and middle areas of the image. Figure 1(b-d) shows the results of image enhancement using traditional HE, CLAHE with a clipping coefficient of 0.01, and GIHE brightness correction methods. Figure 2 is the enlarged image block in Fig. 1.

Figures 1(b-d) and 2(b-d) show the following differences. The traditional HE method involves background over-enhancement, which means that the resulting image appears exceedingly dark overall. The contrast between the protein spots and the background decreases, which degrades the detection and quantitative analysis of protein spots. The CLAHE method with the optimal clipping factor still involves background over-enhancement. The proposed GIHE method enhanced the contrast of weak protein spots against the background.

**Fig. 3:**
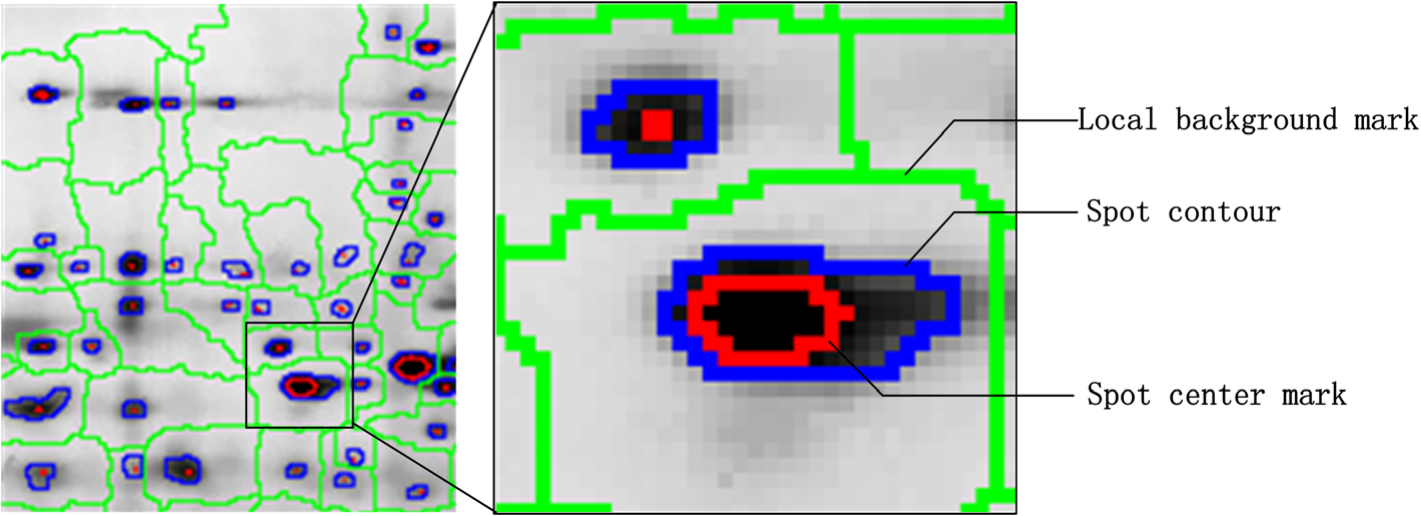
Detection of the spots

To evaluate the enhancement of weak protein spots, we calculated the average gradients of the center and the contour of each weak spot. The spot center was detected with h-dome transformation, and the spot contour was detected with mark-controlled watershed transform. The spot centers and contours are shown in Fig. 3. The weak spots are those with large average gray values and small contrast to the background. In the image of Fig. 1(a), 51 weak spots with average gray values greater than 90 are detected.

**Fig. 4:**
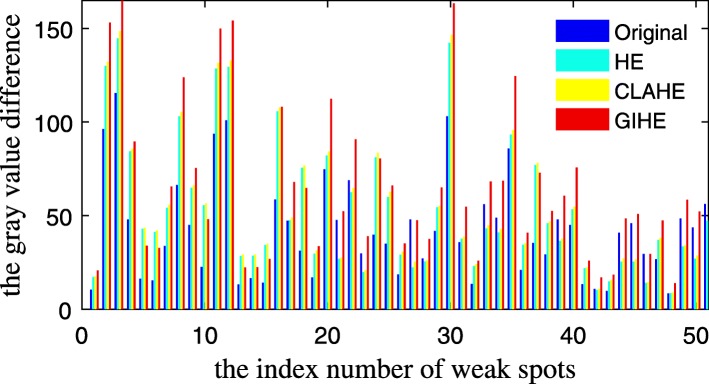
Gray value difference of the weak spots in the 2DE image

The first goal of brightness correction is to reduce the miss rate of detection by enhancing the weak protein spots. Spot detection based on the h-dome transformation depends on the gray value difference between the spot center and background. The gray value difference of the spot can be defined as:

1 $$ d(k)=\frac{1}{M}\sum \limits_{m=1}^M{f}_m^k-\frac{1}{N}\sum \limits_{n=1}^N{f}_n^k,\kern0.75em $$

where *k* is the spot index number, *M* is the number of pixels in the spot center, *N* is the number of pixels in the local background, and *f* is the gray value of the pixels. The gray value differences of the weak spots in the images of Fig. 1(a-d) are shown in Fig. 4.

**Fig. 5:**
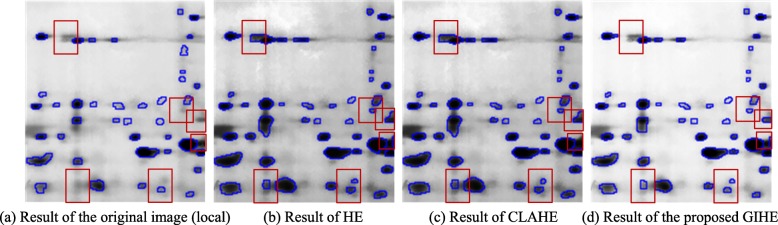
Segmentation results of protein spots in the images in Fig. 1

We can see from Fig. 4 that the HE, CLAHE and GIHE methods all enhanced the gray value differences of weak spots, and the enhancement in the GIHE result image is the largest. Hence, the number of undetected weak spots is reduced. Based on the mark-controlled watershed transform, 553, 564, 569 and 575 protein spots were detected in the images shown in Fig. 1(a-d). The segmentation results are locally shown in Fig. 5.

**Fig. 6:**
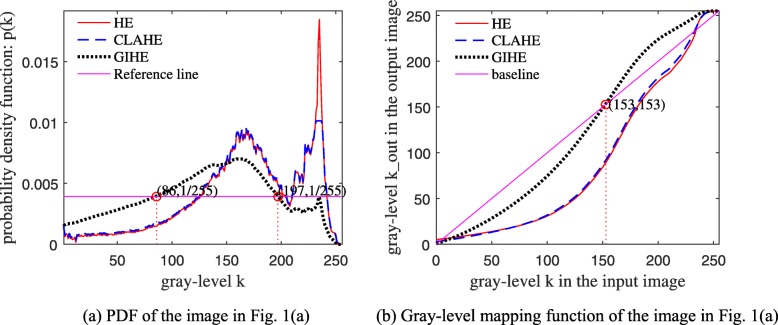
PDF and gray-level mapping function of different algorithms

The effect of brightness correction depends on the gray histogram characteristics. Figure 6(a) shows the PDF of the image in Fig. 1(a). The red, blue and black curves represent the PDFs of HE, CLAHE and the proposed GIHE method, respectively. The PDF of the GIHE method in Fig. 6(a) can be calculated by Eq. (6). Compared to that obtained by HE and CLAHE, the brightness of the result image obtained by the proposed method remains very balanced.

The gray-level mapping function in Eq. (8) is visualized in Fig. 6(b) with the x-axis representing the original gray levels and the y-axis representing the new gray levels. The baseline represents the function *y* = *x*, of which the slope equals 1. From Eq. (8), we know that the slope of the mapping function at the input gray-level *k* is:

2 $$ slope(k)=\frac{255\times c(k)-255\times c\left(k-1\right)}{k-\left(k-1\right)}=255\times p(k) $$

The output gray levels are less than the input gray levels if the curve of the mapping function is below the baseline. From Fig. 6(b), we see that after correction by the proposed GIHE algorithm, the gray levels between 0 and 153 decrease, and the gray levels between 153 and 255 increase.

The contrast of the pixels with gray-level *k* is stretched when *slope*(*k*) > 1, which is equivalent to $p(k)>\frac{1}{255}$ according to Eq. (2). According to the PDF of GIHE, as shown in Fig. 6(a), the gray levels between 86 and 197 satisfy the inequality of $p(k)>\frac{1}{255}$.

**Fig. 7:**
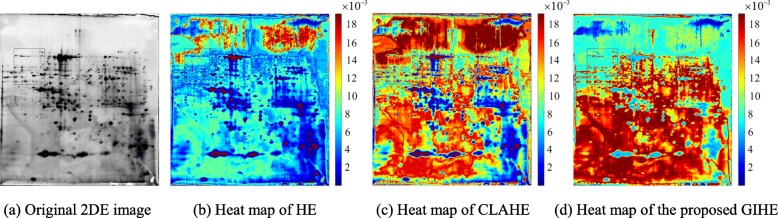
Distribution of contrast enhancement of different algorithms

**Fig. 8:**
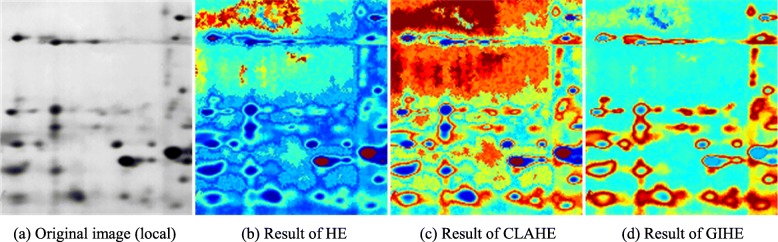
Enlarged image blocks in Fig. 7

In the 2DE image in Fig. 1(a), most of the weak protein spots are distributed in the gray-level interval (86,197), of which the contrast is greatly enhanced. Where are the pixels in the gray-level interval (86,197) located? The two-dimensional PDF matrix, of which each element is the gray-level probability density of each pixel, is visualized as a heat map. The heat maps in Fig. 7 display the distributions of contrast enhancement of the images in Fig. 1. The uppermost color in the color bar represents greater enhancement. Figure 8 is the enlarged display of the image blocks in Fig. 7.

As shown in Fig. 8, HE over-enhances the background and the interior of distinct protein spots. CLAHE enhances the weak protein spots better, and the background remains over-enhanced. GIHE accurately enhances the edge pixels of both weak and distinct protein spots.

### Experiments of 2DE image overall brightness correction based on GIHM

**Fig. 9:**
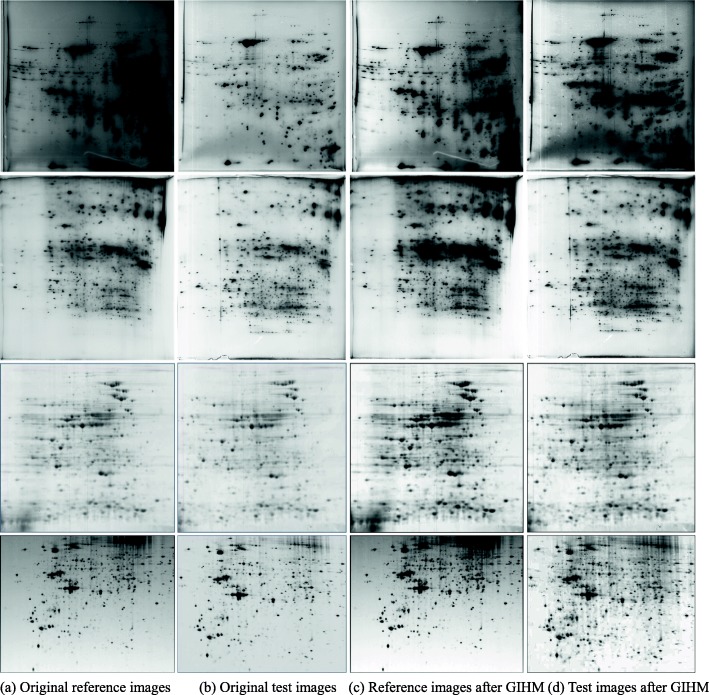
Brightness correction results of real 2DE image pairs with the proposed method

The aim of the GIHM method is to eliminate the overall brightness difference between intra-group 2DE images. To verify the effectiveness of this method, experiments were carried out to correct the brightness of multiple 2DE image pairs to be matched. In Fig. 9, the left two columns are the original 2DE images, and the right two columns are the resulting 2DE images. The images in the upper two rows are from our laboratory, and the images in the lower two rows are from PDQuest test image set.

**Fig. 10:**
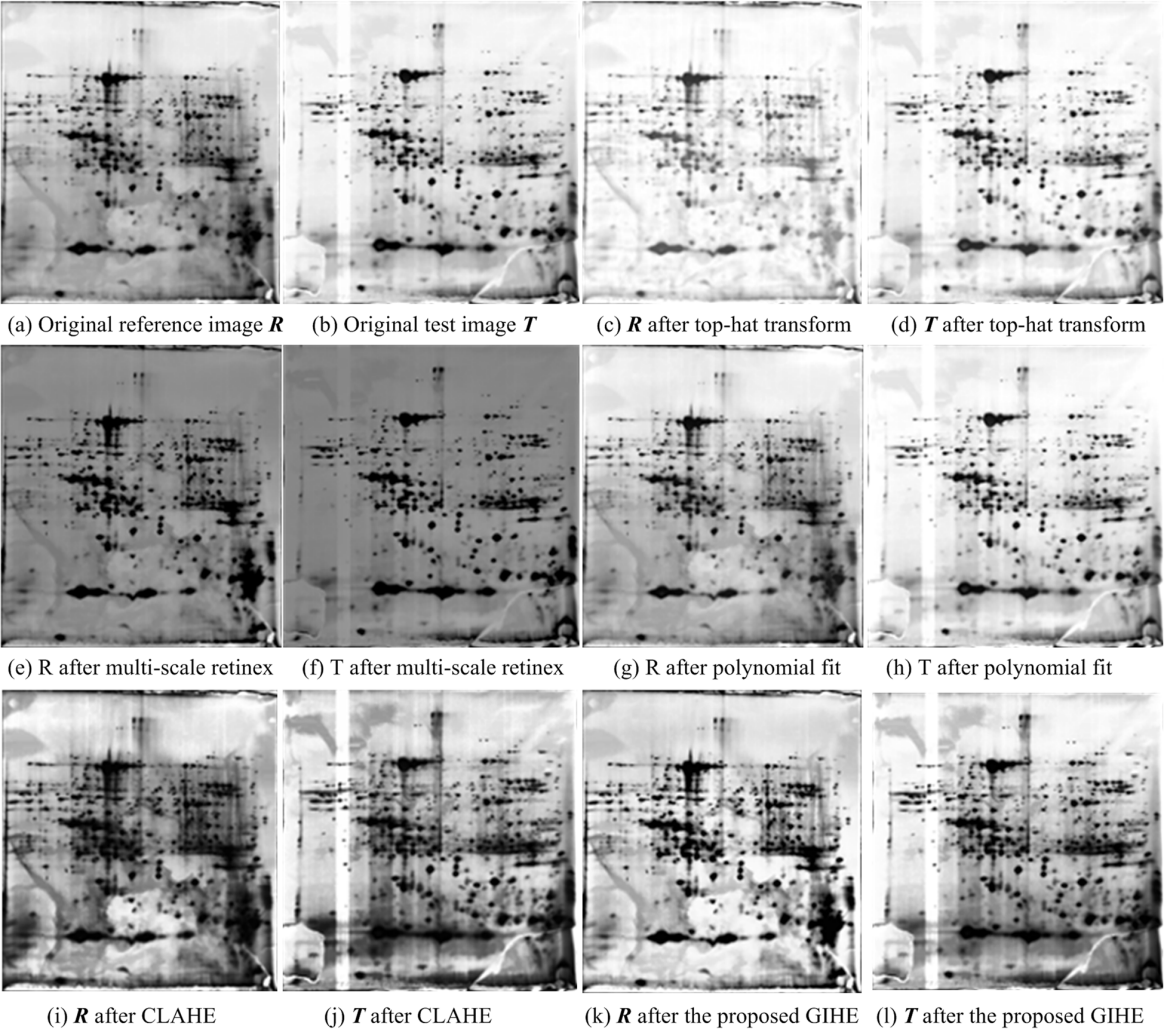
Global brightness shift correction results of the same 2DE image obtained with different methods

As shown in Figs. 9 and 10, there is a great difference between the overall brightness of the original 2DE image pairs, and a large number of small protein spots with low intensities are found in each image. After correction, the 2DE image pairs show basically the same overall brightness, and the contrast of the weak protein spots is significantly enhanced in each image.

Figure 10 shows the results of the overall brightness offset correction for the same 2DE image pair after different methods were used. Figure 10(a) is the original 2DE image pair to be matched, which has a large difference in overall brightness. Figure 10(b) shows the brightness correction results of the top-hat transformation. The image brightness was corrected by reducing the image background with an appropriate structural element. Figure 10(c) shows the results of the CLAHE brightness correction. Figure 10(d) shows the brightness correction results of the proposed GIHM method. The comparison of the results shows that the proposed method performs more accurately in brightness correction and needs no input parameters.

**Fig. 11:**
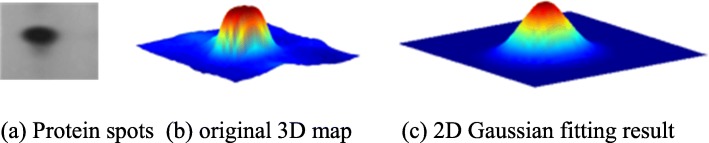
Spot quantification with 2D Gaussian fitting

The aim of brightness correction of 2DE images is to achieve more accurate spot quantification. Common methods for spot quantification are area-based approaches and 2D Gaussian function fitting-based approaches. The two classes of methods have their own advantages, e.g., the compound 2D Gaussian fitting algorithm [18] is the most accurate method for overlapped spots. Figure 11 shows the spot quantification with 2D Gaussian fitting; only seven parameters were needed to describe a spot. This is significant for compressing gel data to build a gel database. However, for weak spots, the model parameters are sensitive to the pixel samples involved in fitting, and the relationship between the parameters of the 2D Gaussian function and the spot brightness is indirect.

For independently analyzing how the pixel intensities are influenced by the brightness correction methods, the area-based method is a more suitable quantification method. Spot volume is the most applied area-based measure and is defined as the sum of pixel intensities within a spot area. The spot quantification depends on the area and the pixel intensities:

3 $$ volume=\sum \limits_{i=1}^N intesit{y}_i=N\times \frac{1}{N}\sum \limits_{i=1}^N intesit{y}_i= area\times \overline{intensity} $$

where *area* is the number of pixels *N* within the spot and $\overline{intensity}$ is the average intensity of the spot pixels.

The corresponding protein spots (CPS) that represent identical proteins in different 2D images are assumed to have the same volume. In fact, different segmentation methods result in different spot boundaries and areas in 2DE images. In this paper, we assume that the areas of the CPS are the same and focus on the intensity factor of spot quantification. Hence, $\overline{intensity}$ (the average intensity of a spot) is used to indicate the quantification.

The intensity ranges of the result images are different. For example, in Fig. 10(c) and (f), the images are first normalized with the image mean and variance:

4 $$ {G}^{\hbox{'}}\left(i,j\right)={M}_0+\left(G\left(i,j\right)-M\right)\sqrt{\frac{V_0}{V}} $$

where both *M*_0_ and *V*_0_ are constants equal to 127 for each image, $M=\frac{1}{H\times W}\sum \limits_{i=1}^H\sum \limits_{j=1}^WG\left(i,j\right)$ and $V=\frac{1}{H\times W}\sum \limits_{i=1}^H\sum \limits_{j=1}^W{\left[G\left(i,j\right)-M\right]}^2$. Then, the quantification error of the *k*th CPS in the reference and test images is:

5 $$ {D}_k=\frac{Q_{A,k}-{Q}_{B,k}}{\raisebox{1ex}{${Q}_{A,k}+{Q}_{B,k}$}\!\left/ \!\raisebox{-1ex}{$2$}\right.}\times 100\% $$

where *Q*_*A*, *k*_ is the average intensity of the *k*th protein spot in image A and *Q*_*B*, *k*_ is the average intensity of the corresponding CPS in image B.

**Fig. 12:**
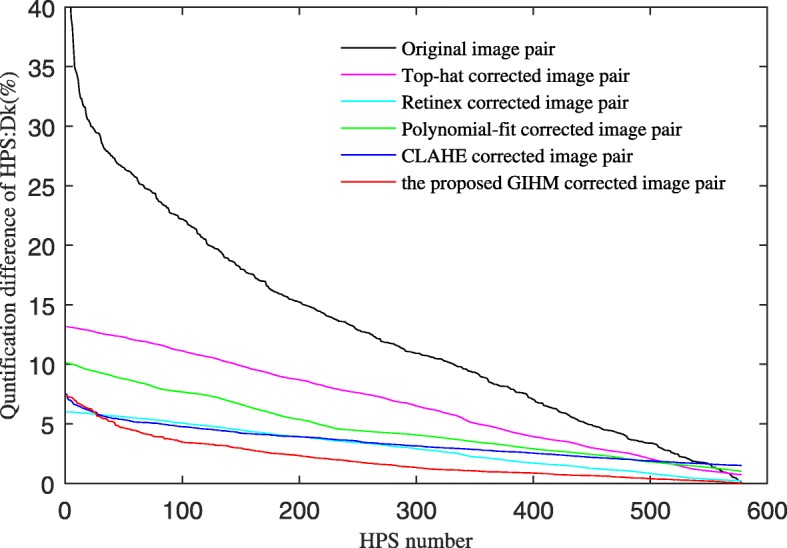
Error of CPS quantification

A total of 578 CPS pairs were detected with mark-controlled watershed transform and manual confirmation in the six image pairs in Fig. 10. The errors of the 578 CPS quantification were calculated according to Eq. (4) and are shown in Fig. 12. The horizontal axis is the *k*th CPS pair with the order of quantitative difference descending. The vertical axis is the quantitative difference *D*_*k*_ of the *k*th CPS pair.

As seen in Fig. 12, the error of the CPS quantification in the original 2DE image pair is the largest. The top-hat transform corrected image is slightly improved on the whole, and even part of the CPS errors increases. The polynomial fit and CLAHE methods further reduced the error. The proposed GIHM method achieved the least error.

The average error of the CPS quantification of each method in Fig. 10 is shown in the first row of Table 1. In addition, according to Eq. (5), the quantification experiments were performed on the brightness correction results of multiple sets of 2DE images. The statistical results are shown in the other rows in Table 1, where the number of CPS in the 2DE images was obtained based on automatic software detection and manual confirmation.

**Table 1:** The average errors of CPS quantification

Experiment number	CPS number	Original image pair	Top-hat corrected image pair	Retinex corrected image pair	Polynomial corrected image pair	CLAHE corrected image pair	GIHM corrected image pair
1	578	12.986%	6.758%	3.020%	4.579%	3.480%	2.334%
2	1074	11.367%	6.548%	2.518%	4.154%	5.102%	2.039%
3	407	6.117%	2.640%	1.924%	5.061%	3.321%	2.159%
4	813	9.743%	4.092%	2.057%	3.305%	3.771%	2.625%
5	792	9.058%	4.732%	2.730%	3.966%	2.493%	1.706%

As shown in Fig. 12 and Table 1, the error of CPS quantification in the original 2DE image pair is relatively large. The top-hat, polynomial fit and CLAHE methods reduced the average errors of CPS quantification to a certain extent. The multi-scale retinex method performed similarly on the average error of CPS quantification, while the 2DE images after retinex correction showed less contrast. The average error of CPS quantification was reduced to less than 3% in the images after brightness correction by the proposed algorithm, which significantly reduced the error of spot quantification in 2DE images.

## Discussions

In this study, we validated GIHE as a method for single 2DE image brightness correction. GIHE accurately enhances the edge pixels of both weak and distinct protein spots by adaptively modify the image histogram, thereby improving the detection rate of weak spots. The key limitation of the analysis is that we have no ground truth regarding how many protein spots there are in the image. The comparison is based on the manual confirmed spots. GIHE is specially designed for 2DE images and thereby performs better than the methods of HE and CLAHE in enhancing protein spots. For general image enhancement, GIHE is probably not as good as CLAHE. In addition, we validated GIHM as a method for multiple 2DE images brightness correction. The errors of the CPS quantification of the resulting image pairs by GIHM are the least. The fundamental reason for the superior performance of GIHM is that the 2DE image pairs have approximately the same intensity distributions.

## Conclusions

A novel image brightness correction method based on GIH is proposed in this paper. GIHE effectively enhances the weak protein spots and avoids over-enhancement of the background in 2DE images. GIHM significantly improves the average error of CPS quantification in 2DE image pairs. Both GIHE and GIHM require no input parameters, thus the proposed method is convenient for practical application.

## Methods

### GIHE-based enhancement of weak protein spots

To achieve the abovementioned two goals of 2DE image brightness correction, we propose a brand new method based on GIH. According to the statistical characteristics of 2DE images, in which the brightness of the background changes slowly and the brightness of the edge of the protein spots changes quickly, the gradient information of pixels within 8-neighborhood is used to adaptively modify the image histogram. By adaptively increasing the probability density of the pixels of weak protein spots, these spots can be effectively enhanced. The detailed description of the proposed GIHE method is as follows.

Step 1: Calculate the GIH. For 2DE images with 256 gray levels, the histogram is initialized as a one-dimensional zero array with *N* = 256. Let *T*(*x*, *y*) denote any pixel in row *y* and column *x* of image *T*; *k*_max_ and *k*_min_ denote the maximal and minimal gray values in the 8-neighborhood respectively. Then, the elements of the histogram with subscripts between *k*_max_ and *k*_min_ are added by 1, that is:

6 $$ gih(k)= gih(k)+1,\kern0.75em 0\le {k}_{\mathrm{min}}\le k\le {k}_{\mathrm{max}}\le 255 $$

Step 2: Calculate the probability density function (PDF):

7 $$ p(k)=\frac{gih(k)}{\sum \limits_{i=0}^{255} gih(i)},\kern0.75em $$

Step 3: Calculate the cumulative distribution function (CDF):

8 $$ c(k)=\sum \limits_{i=0}^kp(i),\kern1em $$

Step 4: Map the gray-level *k* to a new assigned gray-level *k*_*out*_ according to the CDF:

9 $$ {k}_{out}=F(k)=255\times c(k) $$

Map any pixel *T*(*x*, *y*) using Eq. (9) to generate a result image pixel *J*(*x*, *y*):

10 $$ J\left(x,y\right)=255\times \Big(c\left(T\left(x,y\right)\right) $$

The key point of the algorithm is based on the GIH of the pixels, which can adaptively increase the probability densities of the pixels with high gradients in the protein spot areas.

### GIHM-based overall brightness correction of different 2DE images

Intra-group 2DE images mainly consist of CPS and different kinds of interference. There are spots indicate differential protein expression in inter-group 2DE images. Theoretically, the pixels of CPS have the same brightness distribution. In practice, pixel brightness deviation is usually caused by the overall brightness difference, which seriously affects the quantitative analysis of protein spots. In this paper, we use the GIHM method to correct the brightness of different 2DE images.

Let *u* denotes the continuous gray value in the images *I* and *J, p*_*I*_(*u*) and *p*_*J*_(*u*) denote the PDF of images *I* and *J*, respectively. Then, the CDF of image *I* is:

11 $$ CD{F}_I(v)=f(v)={\int}_0^v{p}_I(u) du,\kern0.75em $$

The CDF of image *J* is:

12 $$ CD{F}_J(w)=g(w)={\int}_0^w{p}_J(u) du,\kern0.75em $$

Let *G* denotes the resulting image of *J*. In order to make the images *G* and *I* have the same intensity distributions, we only need to make their CDF be the same:

13 $$ CD{F}_G(v)= CD{F}_I(v) $$

CDF is a monotonically increasing function with a value range of 0 to 1. Given that the gray value *w* in image *J* is mapped into the gray value *v* in the resulting image *G*, *CDF*_*G*_(*v*) = *CDF*_*J*_(*w*) needs to be satisfied. This equation is equivalent to *f*(*v*) = *g*(*w*). Hence the gray value map function is:

14 $$ v={f}^{-1}\left(g(w)\right) $$

For digital images with discrete gray levels, it is impossible to guarantee that *f*(*v*) = *g*(*w*) could be established. Therefore, the mapping method is implemented by using the smallest absolute difference as follows.

Step 1: The CDF of the reference 2DE image *I* is calculated by the discrete form of Eq. (11):

15 $$ {C}_I(k)=\sum \limits_{i=0}^k{p}_I(i) $$

Step 2: The CDF of the test 2DE image *J* to be corrected is calculated:

16 $$ {C}_J(k)=\sum \limits_{i=0}^k{p}_J(i) $$

Step 3: For each element in *C*_*I*_, find an element in *C*_*J*_ to minimize the distance between them:

17 $$ \underset{n}{\arg }\ \min \mid {C}_I(m)-{C}_J(n)\mid $$

Since both *C*_*I*_ and *C*_*J*_ are monotonically increasing sequences, the gray-level mapping is obtained by the above operations, thus ensuring that the relative gray values of the pixels remain unchanged.

**Fig. 13:**
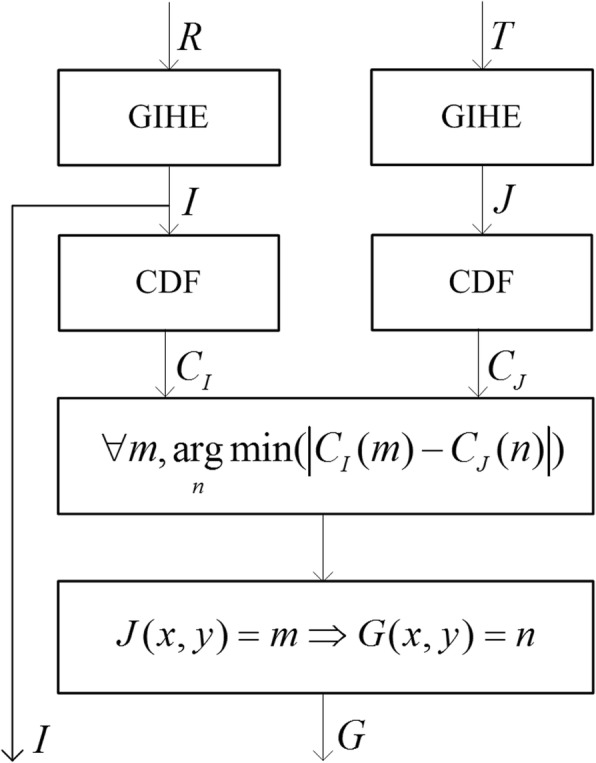
Block diagram of the proposed brightness correction method based on the GIH

For a reference 2DE image *R* and a test image *T* to be matched, the process of brightness correction is shown in Fig. 13.

In Fig. 13, *I* is the resulting image of reference image *R* by GIHE, *J* is the resulting image of test image *T* by GIHE, *C*_*I*_ and *C*_*J*_ represent the cumulative density of images *I* and *J*, respectively. The gray-level *m* in image *J* is mapped into a new value *n* in the resulting image *G* by Eq. (17). The two output images *I* and *G* have similar brightness distributions.

## Data Availability

The original 2DE images have been included in the additional files.

## Abbreviations

2DE: Two dimensional electrophoresis
CDF: Cumulative distribution function
CLAHE: Contrast limited adaptive histogram equalization
CPS: Corresponding protein spots
GIH: Gradient interval histogram
GIHE: Gradient interval histogram equalization
GIHM: Gradient interval histogram match
HE: Histogram equalization
PDF: Probability density function
